# Essay on Tumors in the Palatine Region

**Published:** 1857-07

**Authors:** 

**Affiliations:** Member of the Anatomical Society and the Society of Observation.


### ARTICLE IV.

*Essay on Tumors in the Palatine Region.* By DR. PARMENTIER, Member of the Anatomical Society and the Society of Observation.* Translated from the French-L'Art Dentaire, for the Am. Jour. Dental Science.

THE present work was suggested to me by my excellent friend and instructor, Dr. Demarquay at a time when I was preparing for his course of surgical anatomy. He was so good as to furnish me with numerous materials which he had collected for his own use, and from which I have derived much benefit as will be seen in the course of this treatise.

*Introduction.*—Tumors of the palatine region are interesting to the surgeon, because of the symptoms to which they give rise, and the operations which they necessitate. In effect, they impede the performance of the important functions with which the organs of these parts are associated; thus it becomes necessary to remove them promptly, not only because of the nature which they sometime assume, but because they often obstruct the two functions, digestion and respiration, necessary to the

*Part of this work has already appeared in the Medical Gazette, but the anatomical and physiological portion, forming the two first chapters, has never before appeared; we are the more obliged to Mr. Parmentier for consenting to place it at our disposal, as the surgical and physiological anatomy of the palatine region, is of much importance in the construction of obturators—a department to which we have devoted much attention and which will give us frequent opportunities to entertain our readers. *Eds. L'Art Dentaire.*

preservation of life. Finally, the situation of the region occupied by these morbid productions, the abundance of vessels presented by the anatomy of these parts, render these operations sometimes extremely difficult; hence it becomes necessary for the surgeon in devising the proper method of procedure to tax his ingenuity so as not, in the removal of the disease, to cause hemorrhage which is, more or less, liable to occasion the death of the patient.

Besides, the region of the palate being liberally supplied with nerves, operations on these parts are very painful, and anæsthetics cannot be employed on account of the situation of the field of operation.

When these facts are considered, it seems really astonishing that authors of classical works on pathology silently pass over the history of the tumors of the palatine region, for they have been frequently observed and meagre descriptions only are to be found scattered through the annals of medicine; by the aid of the facts already observed, and some cases which came to my notice during my medical studies, I shall attempt to trace the history of the morbid productions under consideration. But before I do so, it may be proper to give a brief description of the anatomy of the region, noticing the physiological knowledge we posses of the functions of the organs of which it is composed.

### PART FIRST.—*Anatomy and Physiology of the Palatine Region.*

### CHAPTER I.

*Anatomy.*—The palatine region is composed of two parts—the anterior, the vault of the palate; the posterior, the velum palati. This region is clearly defined in front and upon the sides by the dental arch, and behind by the sinuous lower edge of the velum; it is united on each side by a muscular projection known by the name of anterior pillar of the palate, and in the region of the pharnyx by another equally muscular projection, the posterior pillar of the palate. The anterior, fixed and immovable portion separates the entrances of the digestive and respiratory passages. Being placed between these two organs of the senses, taste and olfaction, it constitutes the perfection of the first; it is here that the tongue completes the preparation of alimentary substances taken into the buccal cavity. Thus the anterior portion of the palatine region aids the sense of smell, for after the introduction of alimentary substances they are often immediately rejected. The posterior part, on the contrary, is very movable, constituting the first valve in the digestive and respiratory canals; it directs the alimentary bolus thence into its final entrance into the digestive canal, and gives egress to the expired air which transmits the vibrations of the vocal cords as well through the nostrils as the vocal cavity; this portion of the region of which we are now speaking affects the two nutritive functions, digestion and respiration, as well as that of the voice. In treating of the physiology I shall examine thc action of the vault and velum and the different functions which I shall mention.

Concave from front to back and transversely in its anterior portion, and oblique from top to bottom and front to back in its posterior part.

The frame of the palatine region is bony before and fibrous behind; the osseous portion is formed by the palatine processes of the superior maxillary and the horizontal portion of the palatine bone; it is here that syphilis so frequently produces caries and necrosis, causing communications between the nose and mouth, which we are fortunately able to close by means of properly constructed obturators, but which always affect, in a more or less marked manner, the tone of the voice. When the median suture of the vault does not extend to the back portion of the bones, an abnormal division of the veil of the palate results. This most frequently happens; there are, however, exceptions. M. Demarquay and myself have met with a patient in which the posterior portion of the horizontal plate of the palate bone did not exist but without a separation of the velum; we observed, however, that the palate-staphylinus was wanting. If the division is prolonged anteriorly two conditions may exist. In the one the slit forks and its branches enclose the intermaxillary bone, ordinarily producing double hare-lip. In the other, there is no forking, but the slit extends obliquely forwards, causing simple hare-lip. Finally, it is possible that the slit may extend from one end of the palatine vault to the other without quitting the median line. These cases are ordinarily the result of congenital defects of the fetus, accompanied generally by a weaker development of bone than exist when the formation is perfect. The velum may also present a congenital division which may exist alone or be accompanied by a separation of the palatine suture, prolonging itself anteriorly and terminating in a single or double hare-lip. It is for this defect noticed long ago and treated successfully by suture by Lemonmer, in 1764, but which was regarded as incurable, so that Graef, of Berlin, and Professor Roux invented staphyloraphy, an operation simple and easy of itself, but rendered difficult and tedious by the mobility and depth of the part upon which it is performed.

Professor Velpeau relates, in his Surgical Anatomy, that he had seen a dead subject in which there was no horizontal portion of the palatine bone or palatine process of the superior maxillary. The palatine membrane has double the natural thickness and the density of fibro-cartilage. That of the floor of the nasal cavities presents the same peculiarity. These are separated by a space of a line and a half, occupied by a kind of mucus, forming a closed cavity between the mouth and nasal fossæ.

The solid part of the veil of the palate is formed of a fibrous layer, belonging to the two external peristaphylini muscles. All the muscles of the palatine veil are fixed here, the internal peristaphylinus, the glosso-pharyngeal, and the palato-staphylinus; the last named muscle belongs to the velum in all its extent; the others only terminate there.

The internal peristaphylinus draws the velum towards the nasal fossæ; the external peristaphylinus enlarge it by drawing it horizontally towards the pterygoid apophysis; the pharyngo and glosso-staphylini force it towards the base of the tongue; finally, the palato-staphylini elevate it in the direction of the glottis.

The situation of these muscles easily explain the separation of this organ when it is divided, but we cannot readily account for the reunion of the diseased parts of the palatine veil.

These muscles are separated from the mucous membrane by a laminated bed filled with very large follicles; it is in the thickness of this membrane that purulent œdematous secretions originate. The follicles belong to the species of nucleated glands, their number is considerable in the anterior as well as the posterior surface. On a level with the reflection of the interior of the peristophylini above the hook of the pterygoid apophysis, these glands form a considerable accumulation in front of the point of reflection of the palatine arteries. This mass of glands which furnish a large quantity of mucus when an operation is peformed upon the veil of the palate, protects the deeply situated arteries, and which explains how incisions made in the veil of the palate are rarely followed by hemorrhages. These follicles when hypertrophing cause tumors which project into the mouth as well as into the pharnyx and nasal fossæ; these tumors resemble mammary tumors, to which Mr. Cruveilher has given the name of fibrous species of mammary, and which the microscope of Dr. Lebert has shown to be nothing more than a secretion of one of the mammillary glands; we might anticipate and say that this latter observer has found the same elements in several tumors of the palatine vault which were submitted to his examination as in the mammary secretion, which has been confirmed by the microscope, showing the identity of the nature of the mammary, of the parotid, and of the veil of the palate; I shall have occasion to treat at length of tumors caused by hypertrophy of the follicles of the velum palati.

The anatomical elements described up to the present do not belong, as will be perceived, to the whole extent of the palatine region; those which remain to be noticed are distributed in the vault of the palate and velum. These are the mucous membrane, the vessels and the nerves.

The bones of the anterior part of the palatine region is immediately covered by the mucous membrane which is dense, slightly colored, doubled by a thick fibrous cellular tissue; presenting many hard transverse wrinkles, especially in front; more soft and redder behind, covered by an epithelium easily perceived, which, in many diseases is more or less elevated. It sometimes gives origin to very hard fibrous bodies which rarely acquire a great size in the velum. The mucous membrane is thick, unyielding, easily removed, higher colored than that of the mouth of which it is a continuation in front and of that of the nasal cavities behind. These two membranes connect at the lower margin of the velum; they are united by very loose cellular tissue which becomes infiltrated in inflammation of the fauces. We find here a small appendix, the glottis, which contains in its thickness a large number of follicles. It is the swelling of these, the inflammation of the cellular tissue covering them or the secretion of the layers of the mucous membrane that is commonly called disease of the glottis. Of whatever nature this elongation may be without being considerable, it can prove very troublesome by causing a disagreeable cough by the irritation it keeps up in the tickling of the tongue so as to lead some to believe the existence of a pulmonary disease, and hence many physicians have cited cures by merely removing the uvula. The greater portion of this may be removed without serious inconvenience, as is proved every day by the destruction of it by syphilitic ulcers. It has been frequently thought to have been removed to the root, when in fact only that portion had been taken away below the palato-staphylinus muscle, the remaining tissues resuming their respective positions. As it appears then nearly as long as in its normal state, many surgeons have thought it was regenerated. The point of support which it gives to the velum against the pharyngean wall and the part it plays in singing, renders it impossible to remove a portion of it without exposing secretions entering the nasal cavities from the stomach and without more or less sensibly altering the voice.

The vessels and nerves of the palatine region are very numerous.

The arteries are derived from the superior and inferior palatine and the pharyngeal; in the anterior portion the branches of the superior palatine artery are distributed, which emerge from the posterior palatine foramen, and proceed along the sides of the vault, first, towards the bone and fibrous tissue, then between the fibrous and mucous layers.

M. Delabarre gives an example of an aneurism of one of these arteries; I shall take occasion to speak of aneurism of this region and of enumerating the different methods of treatment which have been proposed.

The veins have the same course as the arteries and empty themselves into the branches, which accompany the internal maxillary artery.

The capillary vessels are very abundant in the veil of the palate which will perhaps explain the advantage resulting from touching this part with nitrate of silver or alum.

The lymphatics empty themselves into the sub-maxillary ganglions and offer no other remarkable peculiarities.

The nerves proceed from the spheno-palatine ganglion and arrive at the region of the palate by two different routes. The posterior, middle, and anterior palatine enter through the posterior palatine foramen; the ramifications of the naso-palatine emerges from the anterior palatine foramen, and proceeds to the mucous membrane of the palate after having traversed a ganglion discovered by M. Cloquet. This ganglion, the existence of which has subsequetly been denied by many anatomists, has been more recently re-established by M. Demarquay. It is situated above the incisive conduit, as may be seen in anatomical preparations deposited in the museum of the faculty.

The glosso and pharyngeo-staphylinus muscles are supplied by fillets from the plexus that forms the glosso-pharyngean on the sides of the pharynx and tongue; the exterior peristaphylinus are supplied by a fibre of the pterygoidean branch of the lower maxillary while the anterior palatine nerves send fillets to the internal peristaphylinus and the palato-peristaphylinus.

After the brief description which I have given of the anatomical elements of the palatine region, we can readily judge of the importance of these parts in a surgical and physiological point of view. M. le Docteur Demarquay in his anatomical and surgical lectures dwells at length upon this small region which owes its importance as much to the number of its blood vessels and nerves as to the position which it occupies—a position which is such that whatever the pathological condition it may take on, an immediate modification of function results therefrom; this latter remark leads me at once to the consideration of the physiology of the vault and veil of the palate.

### CHAPTER II.

*Physiology.*—The anatomical elements of the palate which we have just described, show that this region consists of a fixed and immovable part which can only be employed in the different functions in which this region takes part.

The palatine region plays a part in the prehension and deglutition both of liquids and solids, in respiration, taste, olfaction, phonation and the articulation of words.

Let us examine successively the movements of the palate in all its physiological acts.

### *Prehension of Liquids.*

1. *Deglutition.*—The vault and veil of the palate take part in the two first stages of deglutition; they aid in conveying the alimentary bolus to the isthmus of the pharynx. (First stage.)

They prevent it from entering the nasal cavity and returning into the mouth. (Second stage.)

*First Stage.*—After the alimentary bolus is placed upon the tongue which raises it towards the palatine vault where a point of resistance is offered; but when the bolus arrives at the velum palati, this is expanded by the contraction of the outer peristaphylinus, while the glosso-staphylinus holds it down.

*Second Stage.*—The veil of the palate approaches near to the pharynx and consequently contributes to its elevation by means of the muscle of its posterior pillar which connects with it and the pharynx (pharyngo-staphylinus.) It is by the contraction of the two muscles of this name, muscles, the reunion of which, forms, as has been shown by Professor Gerdy, an oblique sphinctor, dividing the pharynx into two parts: the one superior, which communicates with the nasal fossæ, the other, the inferior, through which passes the alimentary bolus that causes the approximation of the two lips in cases of congenital diversion of the velum palati. MM. Gerdy and Dzondi have denied the contraction of the velum during the second stage of deglutition; nevertheless it does certainly happen as has been demonstrated by MM. Maissiat and Debrow: but there is no inversion of the velum which would apply to the posterior opening of the nasal fossæ; on the contrary, the posterior pillars approaching from an inclined plane from before to behind and from above to below, over which is passed the alimentary bolus.

The elevatory motion of the velum is owing, according to Professor Berard, as much to the raising of this valve by the base of the tongue which is applied at the moment when the isthmus of the pharynx opens, as to the contraction of the elevator muscles. This elevatory movement is instantly succeeded and always during the second stage of deglutition by the depression of the glosso-staphylinus, which assists in preventing the return of the alimentary bolus.

The velum also assists in the prehension of liquids; it is lowered during sucking, drinking, &c., while during the last stage in vomiting as in the second stage of deglutition it obstructs the passage of any thing through the nasal fossæ, sometimes, however, they partially escape here when the action of the pharyngo-staphylinus is incomplete.

2. *Respiration.*—In inspiration through the nostrils the velum palati is lowered by the action of the glosso-staphylinus muscle, which is brought in contact with the base of the tongue which is at the same time raised, thus preventing all communication of the mouth and pharynx. In inspiration through the mouth the veil of the palate and tongue separate and prevent all communication between the nasal fossæ and pharynx.

3. *Taste.*—In the sense of taste the vault of the palate acts in a purely mechanical manner; it furnishes the tongue a solid and rough surface against which it multiplies its points of contact with the savory substance. It is wrong in this case to ascribe one half of the sense of taste to the palate; for substances pass in exactly the same manner when the palate is covered with an impermeable and tasteless pellicle, whilst if the pellicle is applied to the tongue and the savory substance laid upon it and the action repeated, no taste whatever can be perceived.

The velum of the palate in the centre of its anterior surface is active in gustation, but for the degree of delicacy and its susceptibility of being impressed with savors, it occupies the last place amongst gustative surfaces.

4. *Olfaction.*—There are circumstances in which it is to our advantage to diminish the olfactory sensations; as for example, as soon as a disagreeable odor has reached us, we endeavor by a strong expiration to expel the odorous air, then inspiration instead of being carried on through the nostrils is effected through the mouth; the velum of the palate raises itself horizontally, closing the posterior opening of the nasal fossæ, preventing the circulation of air in their interior, and consequently preventing the return of disagreeable impressions to the olfactory membrane. Supported by these observations and analogy in the way of nervous repartition.* M. Longet has been led to deduce a physiological relation between the iris and velum of the palate; that is to say, in the latter a method by which we may protect ourselves against disagreeable odors, whilst the iris by contracting guards us against too strong a light.

5. *Phonation and Pronunciation.*—The vault and veil of the palate are organs which aid phonation only in an accessory manner; they prevent the admission of a larger quantity of air to the nasal fossæ than can pass through it; but they play an important part in pronunciation when the velum successively assumes different positions according to the letters which are to be articulated.

### CHAPTER III.

*History.*—Thomas Bartholin reports that a lady from Denmark, having had a fluxion from the left side of the upper jaw, was followed by the development of a painful tumor, which having suppurated a stone as large as a small walnut came away.†

The same author says in his Anatomy, that he has often seen calculi in the vault of the palate.

* The nerves, in effect, do not reach the iris and velum palate until after having transversed a ganglion.

† This case is quoted by Jourdain in his treatise on the diseases of the mouth.

Ruysch reports two cases of fungosities of the palate accompanied by caries of a portion of the bones of the vault; he treated successfully one of these cases, by removing the fungus with a bistoury and cauterizing the carious bone; but the subject of the other cases would not submit to the treatment which he proposed, and consequently died.

In his Surgical Magazine, Scultet, relates that he was consulted by a woman who had had for three months, on a level with the anterior palatine foramen, excrescences which bled profusely from the slightest touch of the tongue. He touched the tumor with a probe which made it bleed very much, when he perceived that it had its origin in the anterior palatine foramen; he destroyed it by excision and cauterization.

Kruger, Med. Jour. vol. 5th, page 69, speaks of an abscess of the palate, which when it broke, was found to contain a large calculus of an ashy color and compact texture.

In the *Journal of Medicine* of Vandermound, vol. xiii, page 433, we find the history of the extirpation of a tumor of the palate by Anselin, surgeon at Amiens. The removal of this tumor which was of a fibrous nature, was followed by hemorrhage which was easily suppressed by means of a compress.

In the 36th vol. of the Journal, page 269, year 1771, Botot relates the history of a case of a tumor of the vault of the palate which he removed by extirpation with a cutting instrument.

In 1772, Jourdain published in the 37th vol. of the Journal of Medicine, a memoir upon the diseases of the palate, many cases of tumor of the palatine vault.

In 1778, the same author published his treatise upon the diseases of the mouth, in which he has devoted a long chapter to the diseases of the palate, particularly tumors; we find there many references to authors who have written upon the subject, and besides these he notices other cases which came under his his own observation; I shall notice these in the next chapter.

In the Hospital Gazette of 1844, page 434, we find a note from Dr. Charsaignac upon medico-palatine exostosis.

In 1844, page 290, the same journal gives an account of cancerous tumor taken from the practice of Professor Blandin; this is the first case of tumor of this region, subjected to microscopical examination.

Demarquay, in 1845, published an account of a remarkable case of tumor of the velum palati, which Professor Blandin removed by ligature; this article is headed, *cancer of the veil of the palate.* I shall show, in speaking of cancerous tumors, that this should not be considered as such; the knowledge which I have obtained in the hospital of the disease itself confirms me in this opinion.

In the *Dictionary of Medicine*, vol. 30, article *veil of the palate*, (pathology,) page 833, P. Ollivier, only mentions in a passing manner, cancer of this organ, and says it may be destroyed by cauterization, extirpation and ligature; he adds in conclusion, in a case where caustics had been unsuccessfully employed by Professor Blandin, and in which he feared hemorrhage after excision, he had recourse to a particular kind of ligature.

Dr. Mayolin had occasion to remove, in 1851, a tumor situated in the thickness of the veil of the palate; it is said that the disease had a cancerous aspect.

Two years before, Dr. Chassaignac made the resection of the superior maxillary for the removal of a large cancer of the palatine region. *

The annals of surgery furnish numerous examples, proving the existence of varicose bloody tumors seated upon the cheeks, lips, eyelids, forehead, occiput and the ears, but we know of but one case of congenital varicose sanguinous tumor situated in the interior of the mouth; it has been observed by Professor Scarpa, that it developed itself as a varicose sanguinous tumor in the roof of the mouth.

Dr. Loir presented to the clinique of M. Dupuytren, an osseous cyst, developed from the palatine apophysis of the left superior maxillary bone, the walls of which were formed by the two compact layers of this apophysis, the immediate cause was evidently a misplaced tooth. In effect, the left canine tooth

* Hospital Gazette, 1849, page 409.

had opened a passage in the internal wall of this bone, and had caused a cavity three times the size it would have done upon the exterior, in the cellular tissue of the palatine apophysis where it was developed. The root of this tooth formed an abutting arch against the external wall of the alveolar border.

M. Laborie presented to the Anatomical Society, a syphilitic excrescence developed upon the extremity of the uvula. This tumor had a pedicle of five or six lines in length, supporting a granulated excrescence, of a cauliflower shape, of the size of a large pea. This excrescence, which was of a livid color, obstructed respiration. It was removed and the patient got well.

But there is a tumor of the velum palati, of a peculiar nature, which has not until very recently attracted the attention of surgeons: it results from hypertrophy of the palatine glands. Professor Nelaton saw it for the first time, eight or nine years ago. Since then, another case has been observed at the Hospital of the Faculty of Medicine. In 1846, M. Marchal (of Calvi) has related a case in the Hospital Gazette.

In 1851, M. Michon saw a case in a man thirty-six years of age.

### CHAPTER IV.

*Description of Tumors in the Palatine Region.*—Tumors of the palatine region may be divided into liquid and solid.

In section first, we shall notice abscesses, gummy tumors, serous cysts, and sanguinous tumors.

In the second section we will treat hypertrophy of the glands of the velum palate, exostosis, bony cysts, fibrous and carcinomatous tumors.

*First Section—Liquid Tumors—Abscess.*—Abscess develops itself as often in the vault of the palate, as in the velum; again, it appears at the same time in both parts of the palatine region.

As in all parts of the body, these abscesses result sometimes from simple inflammation of the cellular tissue, at other times they are symptomatic of disease of the osseous tissue of the mouth.

Abscesses arising from inflammation of the cellular tissue, occur both in the vault and veil of the palate; the abscess of which Kruger speaks, Journal of Medicine, vol. 5, page 59, and noticed in Jourdain's Treatise on the Diseases of the Mouth, was situated in the back part of the vault and upper part of the velum palati; that of which Thomas Bartholin speaks, chap. 5, page 91, was situated in the left side of the vault of the palate; the one which I saw in 1852, while with Professor Malgaigne, was situated in the upper part of the anterior pillar of the velum. These abscesses often appear without any appreciable cause; at other times they succeed to the extraction of a tooth, but such occur as a consequence of lesion of the bone;* we have known them to result from the pricking of fishbones. Dr. Demarquay has seen many abscesses which had resulted from artificial dentures constructed from hippopotamus ivory. These abscesses are particularly the result of this method of prosthesis.†

The diseases under consideration arise more frequently from caries of the alveolar processes and palatine bones, than from any other cause. Abscess of the velum is attended by pain in the fauces, and a sensation that there is something there to be swallowed. At such times it is impossible to swallow the saliva, as pain is at the same time felt in the corresponding ear, the the mouth is opened with difficulty, and when an attempt is made to swallow liquids, they often escape into the nasal fossæ, and to prevent their egress from these passages it is necessary to compress the nostrils with the thumb and fingers; the voice is also rendered thick and unintelligible. Fever, want of appetite, and more or less of keen thirst is experienced; at the expiration of several days, suppuration will have taken place in the centre of the tumor, when if touched, fluctuation will be perceived.

When the abscess succeeds to the extraction of a tooth, it is accompanied by swelling of the face; the pus which often

* See Jourdain Jour. de Med., vol. 37, year 1772, p. 542, obs. i.

† Jourdain loc. cit., obs. iv.

forms at the point where the bone is diseased, frequently traverses underneath the mucous membrane of the palate, so that when the abscess is opened, either spontaneously or by art, the bone will be found more or less denuded.

Symptomatic abscess of the palatine vault is first manifested by swelling of the face, nose and lip, a tumor then appears in the roof of the mouth, which ultimately opens of itself, if not lanced by the surgeon; the opening remains fistulous, fungous excrescences are developed, which attain greater or less size.

As soon as the presence of pus can be detected, an opening should be made to prevent the spreading of the matter, and the separation of the mucous membrane from the superjacent bones, which would be followed by superficial necrosis and exfoliation of part of the osseous tissue. The pus which escapes from the abscess is often as fetid as that which comes from the natural openings.

I have already mentioned the two abscesses noticed by Bartholin and Kruger, in which calculi were found. I saw, in 1852, while in the office of Professor Malgaigne, a woman who had an abscess in her left arm; upon opening it, pus escaped, and in the centre of the sac, several hard calculi were found of a stony appearance. They were found on being submitted to chemical analysis, to be composed of carbonate of chalk.

After the abscess has been opened, all the symptoms determined by the presence of pus soon ceases, and the wound begins to cicatrise; during the curative process the use of an emolient gargle should be prescribed; but when the abscess is symptomatic of disease of the bone, the opening becomes fistulous, bloody fungous granulations are developed, and serous matter is continually discharged, which continues until the bone is healed.

In cases of this kind it is necessary to incise the walls of the fistulous opening, and thus remove the fungosities, and to cauterize the carious bone with a view of inducing exfoliation; if the bone is in a necrosed condition, the dead portion should be removed with forceps, chisel and mallet.

Finally, if a decayed tooth exist, it should at once be removed. In this operation the palatine artery is sometimes wounded, an accident which seems to have happened to Meeckren, a physician who treated the 23d case described in the work of Jourdain, as well as to this surgeon himself.

The hemorrhage may always be controled by using a compress held in place by a small plate provided with a small tourniquet, a kind of compressor, invented by Auselin, and modified by Jourdain; the latter much prefers compression to cauterization in cases of hemorrhage, because when the eschar is removed the hemorrhage frequently recurs; this happened to a patient who was the subject of the 24th case narrated in his work.

[To be continued.]

